# Clinical whole genome sequencing as a first-tier test at a resource-limited dysmorphology clinic in Mexico

**DOI:** 10.1038/s41525-018-0076-1

**Published:** 2019-02-14

**Authors:** Alicia Scocchia, Kristen M. Wigby, Diane Masser-Frye, Miguel Del Campo, Carolina I. Galarreta, Erin Thorpe, Julia McEachern, Keisha Robinson, Andrew Gross, ICSL Interpretation and Reporting Team, Maren Bennett, Krista Bluske, Carolyn M. Brown, Amanda Buchanan, Brendan Burns, Nicole J. Burns, Anjana Chandrasekhar, Aditi Chawla, Amanda R. Clause, Alison J. Coffey, Maria Laura Cremona, Vlad Gainullin, R. Tanner Hagelstrom, Alka Malhotra, Maya Rajan, Revathi Rajkumar, Sarah Schmidt, Subramanian S. Ajay, Vani Rajan, Denise L. Perry, John W. Belmont, David R. Bentley, Marilyn C. Jones, Ryan J. Taft

**Affiliations:** 1 Illumina, Inc, San Diego, CA 92122 USA; 2 Rady’s Children’s Hospital, San Diego, CA 92123 USA; 3 University of California, San Diego, CA 92093 USA

## Abstract

Patients with rare, undiagnosed, or genetic disease (RUGD) often undergo years of serial testing, commonly referred to as the “diagnostic odyssey”. Patients in resource-limited areas face even greater challenges—a definitive diagnosis may never be reached due to difficulties in gaining access to clinicians, appropriate specialists, and diagnostic testing. Here, we report on a collaboration of the Illumina iHope Program with the Foundation for the Children of the Californias and Hospital Infantil de Las Californias, to enable deployment of clinical whole genome sequencing (cWGS) as first-tier test in a resource-limited dysmorphology clinic in northern Mexico. A total of 60 probands who were followed for a suspected genetic diagnosis and clinically unresolved after expert examination were tested with cWGS, and the ordering clinicians completed a semi-structured survey to investigate change in clinical management resulting from cWGS findings. Clinically significant genomic findings were identified in 68.3% (*n* = 41) of probands. No recurrent molecular diagnoses were observed. Copy number variants or gross chromosomal abnormalities accounted for 48.8% (*n* = 20) of the diagnosed cases, including a mosaic trisomy and suspected derivative chromosomes. A qualitative assessment of clinical management revealed 48.8% (*n* = 20) of those diagnosed had a change in clinical course based on their cWGS results, despite resource limitations. These data suggest that a cWGS first-tier testing approach can benefit patients with suspected genetic disorders.

## Pediatrics: Philanthropic clinical whole-genome sequencing program helps children in Mexico

Whole-genome sequencing (WGS) provides a valuable first-tier diagnostic test at pediatric clinics in resource-limited parts of the world, according to a study of children with suspected genetic disease treated in northern Mexico. A team led by Marilyn Jones from the Rady Children’s Hospital and Ryan Taft from Illumina Inc., both in San Diego, California, USA, describe a collaboration with a volunteer-led clinic in Tijuana, Mexico, where they offered genome sequencing for children with suspected genetic conditions—philanthropically through the iHope Program. Among the 60 families that participated, the clinical laboratory team identified genomic variants with diagnostic relevance in 41 (68%) cases. The genomic information contributed to changes in clinical management for 20 of these children, demonstrating the impact of WGS in places where patients generally don’t have access to medical specialists or other sophisticated molecular tests.

## Introduction

There are estimated to be more than 200 million people worldwide with an unresolved rare genetic disease.^1^ An additional ~4% of births worldwide per year will be affected by a genetic disorder.^2^ Genetic disease is tied to substantial healthcare cost and lost productivity burdens,^3^ although the relative impact is expected to be higher in resource-limited geographies with disadvantaged access to care. A historical analysis of global hemoglobin disorder screening indicates that genetic disorders only become a priority once a country achieves an infant mortality rate of less than 40 per 1000 live births,^4^ suggesting that as the mortality associated with communicable diseases, poor nutrition, and lack of resources declines the relative burden of genetic diseases increases. In resource-rich geographies, pediatric patients with a genetic disorder can account for more than a third of hospital admissions and more than 50% of total charges.^5^ The only systematic survey of genetic disease in a large Mexican hospital showed similar results: more than a third of admissions were associated with a genetic disease, and these patients had more frequent and longer hospital stays and an increased number of surgeries.^5,6^

Clinical genetic assessment of patients with suspected genetic disorders is fundamental to subsequent treatment, but interrogating across clinically heterogeneous phenotypes often involves a process of serial testing for specific conditions. This strategy can be expensive and time-consuming, fostering the possibility of incomplete testing and failure to achieve a molecular diagnosis in many patients.^7^ Clinical whole genome sequencing (cWGS) holds promise as a singular testing platform that allows for the simultaneous interrogation of known disease genes and detection of single nucleotide variants (SNVs), small insertions and/or deletions (indels), copy number variants (CNVs), and some structural chromosomal anomalies.^7–10^ Evidence is accumulating to support the use of cWGS as a first-tier test for patients with suspected Mendelian or chromosomal disease where diagnosis is not possible from clinical examination alone.^7,10,11^

Here, we report on a cohort receiving cWGS through a partnership between Illumina’s iHope Program and the Foundation for the Children of the Californias (US-based 501c3) which supports Hospital Infantil de Las Californias, a non-profit, resource-limited pediatric facility in Baja California, Mexico. For many patients seen at this facility, access to multiple specialists and serial molecular testing to obtain diagnoses is typically not logistically or financially feasible. These underserved pediatric patients represent a unique population in which the effectiveness of cWGS as a first-tier test can be assessed.

## Results

**Fig. 1:**
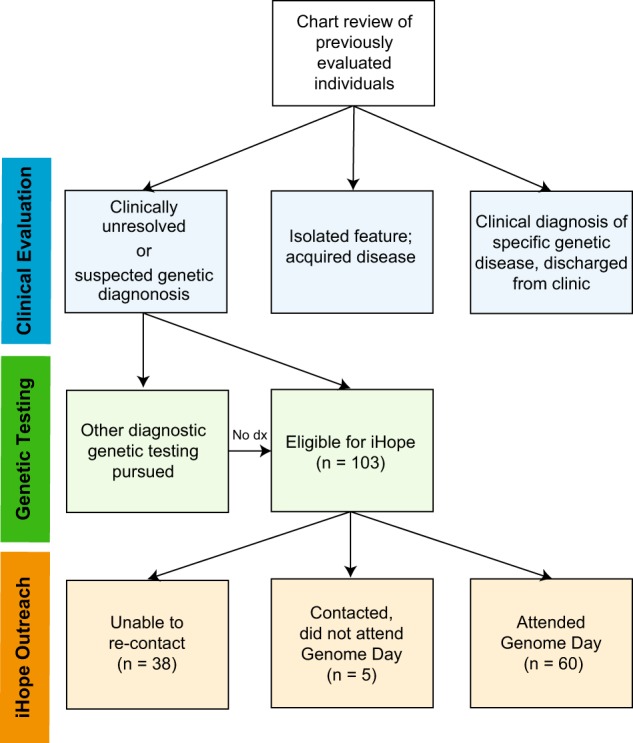
Case selection criteria. Chart review of previously evaluated individuals was performed by the clinician team. Probands who were diagnosed with a recognizable pattern of malformation (e.g., isolated Down syndrome), received counseling, and discharged from clinic were excluded from referral to the iHope Program. Probands with acquired disease (e.g.,: suspected environmental exposures) or isolated features (e.g.,: individuals with cleft lip with or without cleft palate) were typically also excluded. Probands with prior non-diagnostic molecular or cytogenic testing were included if all other criteria were met. Resulting families who were eligible for the iHope Program were contacted, offered cWGS, and scheduled to attend a Genome Day. Upon completion of a Genome Day visit, whole-blood samples were transported to the clinical laboratory for cWGS. Dx: diagnosis; cWGS: clinical whole genome sequencing

Of 103 families deemed eligible for the iHope Program after chart review, sixty probands (58.3%) were successfully contacted and attended a “Genome Day”, a clinic visit dedicated to cWGS testing consent and phenotyping. Of those individuals who did not participate, the majority was due to inability to re-contact the family to offer cWGS (*n* = 38; 79%). The remaining five families were successfully contacted and initially expressed interest in cWGS but did not follow-up with a visit to clinic for a Genome Day (Fig. 1). Six Genome Days were held between August 2016 and March 2018 in which 60 probands and their families (one proband-only, 14 duos, 42 trios, and three quads) provided informed consent and blood samples for cWGS (Table 1). No families who attended a Genome Day and participated in the informed consent process declined to participate in cWGS testing. The mean rounded proband age was 7.6 years at the time of blood draw (ranging from four months to 21 years). Most (*n* = 41; 68.3%) probands had no prior cytogenetic or molecular genetic testing. Of the probands that underwent some genetic testing prior to cWGS, karyotype analysis was the most commonly completed test (*n* = 19). Three probands who underwent karyotype analysis also pursued other genetic testing, including single gene analysis (*n* = 1), methylation studies to assess for Prader-Willi syndrome (*n* = 1), and chromosomal microarray (*n* = 1). The most common indications for testing included congenital anomalies, developmental delay, seizures/epilepsy, growth restriction, and intellectual disability, with clinician categorization showing 76.7% (46/60) of proband phenotypes were consistent with a suspected pattern of malformation and 23.3% (14/60) with a primary neurologic presentation.

**Table 1:** Cohort demographic data

	Cohort (*n* = 60)	
	*n*	%
**Sex** (male)	30	50.0
**Age**		
Birth—2 years	11	18.3
3–8 years	25	41.7
9–14 years	20	33.3
15–21 years	4	6.7
**Phenotype**		
Neurological presentation	14	23.3
Pattern of malformation	46	76.7
**cWGS** **analysis type**		
Proband-only	1	1.7
Duo	14	23.3
Trio	42	70.0
Quad	3	5.0
**Prior genetic testing**		
Karyotype	19	31.7
(+) Single gene testing	(1)	
(+) PWS methylation studies	(1)	
(+) Chromosomal microarray	(1)	
None	41	68.3
**Genome day**		
1 (Aug 2016)	7	11.7
2 (Nov 2016)	10	16.7
3 (Jan 2017)	8	13.3
4 (June 2017)	8	13.3
5 (September 2017)	14	23.3
6 (March 2018)	13	21.7

A genomic finding congruent with the reported phenotype was identified in 41 of 60 probands, resulting in an overall diagnostic yield of 68.3% (Table 2). In 36 probands, pathogenic or likely pathogenic variants were reported in genes or regions of the genome with significant phenotypic overlap, which were considered positive molecular diagnoses. Three probands were considered to have likely positive molecular diagnoses, where variants of unknown significance (VUS) were reported in well-characterized genes, which were confirmed to contribute to the proband’s diagnosis by subsequent clinical feedback (P6, P20, P36). Two cases were considered partial molecular diagnoses, as the identified pathogenic variant was hypothesized to explain the proband’s phenotype only in part (P17 and P31). Of note, molecular diagnoses were achieved for 80% (12/15) of all duo and proband only cases. A diagnostic result from cWGS was obtained for 76.1% of all probands in the patterns of malformation phenotype group compared with 42.9% of all probands in the primary neurologic phenotype group (*p* = 0.0455). All individuals in this cohort received analysis for ACMG secondary findings, and pathogenic secondary findings were reported in three cases (P6, P12, and P39). These include one maternally inherited pathogenic *BRCA2* variant identified in a trio case, one de novo pathogenic deletion encompassing the entire *STK11* gene in a trio case, and one pathogenic deletion involving exons 1–10 of the *PMS2* gene in the proband but not in the proband’s mother identified in a duo case.

**Table 2:** Molecular diagnoses reported by variant type

SNVs and indels						
ID	Age/gender	Family structure^a^	Phenotype	Genomic variant(s) {molecular state-inheritance}	Associated condition (phenotype MIM number)	Classification^b^
P1	15.5-yo/F	Proband-only	Fatigue, muscle pain, exercise intolerance, abnormal gait- walks leaning forward and drags one foot, frequent falls, asthma, short stature, ptosis, short 5th metacarpals, 5th finger clinodactyly, learning difficulties, aggressive behavior, anxiety, amenorrhea, non-specific abnormal muscle biopsy	CHRNE c.1116_1128delGTCGT CGGTGGGC (p.Ser373TyrfsTer8) {hom-unk} [NM_000080.3]	Congenital myasthenic syndrome (605809); AR	LP
P2	5-yo/M	Duo	DD, severe growth restriction with microcephaly, dysmorphic facies, cortical blindness, constant movement	TSEN54 c.919G>T (p.Ala307Ser) {hom-mat} [NM_207346.2]	Pontocerebellar hypoplasia (277470); AR	P
P3	11-yo/F	Duo	Developmental regression, loss of language, purposeful and repetitive hand movement, scoliosis, mildly ataxic gait, self-injurious behavior	MECP2 c.916C>T p.Arg306Cys {het-unk} [NM_004992.3]	Rett syndrome (312750); AD	P
P4	7.5-yo/M	Duo	DD including speech delay, learning and language impairment, hypotonia, camptodactyly, midface hypoplasia, tubular nose, small mouth, secondary alveolar hyperplasia, abnormal palmar creases	NEB c.7645-1G>C {het-mat} [NM_001271208.1] NEB c.11628G>A (p.Trp3876Ter) {het-unk} [NM_001271208.1]	Nemaline myopathy (256030); AR	LP LP
P5	11.5-yo/M	Duo	DD, ID, expressive speech delay involving regression, short stature, right unilateral cryptorchidism, friendly demeanor with plenty of affection, anxiety	KDM5C c.4006dupC (p.Leu1336ProfsTer11) {hem-DN} [NM_004187.3]	X-linked intellectual disability (300534); XL	LP
P6	12-yo/M	Duo	ID, dysmorphic facies, short fourth metatarsals, obesity, and ADHD	EP300 c.2876G>T (p.Ser959Ile) {het-unk} [NM_001429.3] Secondary finding: 749 kb loss at 7p22.1, including at least exons 1–10 of PMS2 (chr7:6027017-6776186) {het-unk}	Rubinstein–Taybi syndrome (613684); AD Lynch syndrome (614337); AD	VUS LP
P7	9.5-yo/M	Trio	Global DD, microcephaly, seizures, truncal hypotonia, choreoathetoid movements, hand-wringing behaviors, sleep disturbance, dysmorphic facies	FOXG1 c.506delG (p.Gly169AlafsTer23) {het-DN} [NM_005249.4]	Rett-like syndrome (613454); AD	P
P8	5-yo/F	Trio	MRI findings of lissencephaly, agenesis of the corpus callosum, small cerebellum with Dandy–Walker malformation. Severe post-natal growth restriction with microcephaly, global DD, ASD, hearing loss, dysmorphic facies, seizures, scoliosis, hypertonia, spastic quadriplegia	TUBA1A c.1096G>C (p.Gly366Arg) {het-DN} [NM_006009.3]	Lissencephaly (611603); AD	P
P9	13-yo/F	Trio	ID, DD, hypotonia, hemivertebrae, scoliosis, long tapered fingers, broad thumbs, and great toes, hallux valgus, broad columella, tooth abnormalities, and relative prognathism	CTNNB1 c.865_869delACAAA (p.Thr289CysfsTer2) {het-DN} [NM_001904.3]	Syndromic intellectual disability (615075); AD	P
P10	4.5-yo/F	Trio	DD, ID, pre- and post-natal growth deficiency, microcephaly, microtia, atresia of the right ear canal, Pierre-Robin sequence with cleft palate, bilateral ear tags, strabismus, proximal placement of thumbs	EFTUD2 c.1904dupT (p.Tyr636LeufsTer8) {het-DN} [NM_ 004247.3]	Mandibulofacial dysostosis- microcephaly syndrome (610536); AD	P
P11	7-yo/M	Trio	Short stature, severe hip dysplasia with flattening and obliteration of the epiphysis, a small fragmented proximal humeral epiphysis, an increased span to height ratio (1.06), an increased upper to lower segment ratio (1.4), limited elbow extension, asymmetric pectus carinatum, lumbar lordosis, umbilical hernia, tapering calves	COL2A1 c.3121G>A (p.Gly1041Ser) {het- DN} [NM_001844.4]	Type II collagenopathies (120140); AD	P
P12	4.5-yo/M	Trio	DD, FTT, microcephaly, recurrent infections, sleep apnea, bilateral eye abnormalities including blepharophimosis and epicanthal folds, cup-shaped ears with a right ear tag, smooth philtrum, bilateral single transverse palmar creases and hypoplastic thenar creases, diastasis recti, hydrocele, pigmentary abnormalities	UBE3B c.518C>A (p.Ser173Ter) {het-mat} [NM_130466.3] UBE3B c.61G>T (p.Glu21Ter) {het-pat} [NM_130466.3] Secondary finding: BRCA2 c.658_659delGT (p.Val220IlefsTer4) {het-mat} [NM_000059.3]	Kaufman oculocerebrofacial syndrome (244450); AR Hereditary breast and ovarian cancer syndrome (612555); AD	P P P
P13	4.5-yo/M	Trio	Striking ataxia, epilepsy, regression of speech and motor ability with onset around age 3	TPP1 c.379C>T (p.Arg127Ter) {het-mat} [NM_000391.3] TPP1 c.1496dupC (p.Leu500SerfsTer18) {het-pat} [NM_000391.3]	Neuronal ceroid lipofuscinosis disease (204500); AR	P P
P14	3-yo/M	Trio	Psychomotor delay, speech delay, low anterior hairline, minor plagiocephaly, broad nasal tip, mild 5th finger clinodactyly, shawl scrotum, inappropriate laughter, wide-based gait	NAA15 c.382C>T (p.Arg128Ter) {het-DN} [NM_057175.3]	Intellectual disability (617787); AD	P
P15	17.5-yo/M	Trio	DD, ID, growth delay, mild microcephaly, dysmorphic facies, low hair line, short neck, decrease elbow mobility, prominent fetal pads, cryptorchidism, constipation	KMT2A c.3034C>T (p.Gln1012Ter) {het- DN} [NM_001197104.1]	Wiedemann Steiner syndrome (605130); AD	P
P16	21.5-yo/F	Trio	ID, expressive language delay, hydronephrosis, bilateral hip dysplasia, septal hypertrophy and cardiomyopathy, coarse facies, maxillary hypoplasia, thick curly hair, hypoplastic distal phalanges with thick knuckles and small nails, redundant and loose skin on hands	HRAS c.34G>A (p.Gly12Ser) {het-DN} [NM_005343.2]	Costello syndrome (218040); AD	P
P17	1.5-yo/F	Trio	Epilepsy, appendicular hypertonia, axial hypotonia, feeding difficulties, constipation, short sternum, one adducted and short thumb, clinodactyly of the second fingers and second toes, syndactyly on both hands, absence of distal creases	CACNA1G c.4591A>G (p.Met1531Val) {het-DN} [NM_018896.4]	CACNA1G-related Disorders (616795); AD	LP
P18	12-yo/F	Trio	Global DD with absent speech, behavioral issues, feeding difficulties, post-natal growth retardation with microcephaly, seizures, ASD	SMARCA4 c.2678T>G (p.Leu893Arg) {het-DN} [NM_001128849.1]	Coffin-Siris syndrome (614609); AD	LP
P19	16-yo/F	Trio	ID, DD (motor and speech delay with progressive selective mutism), self- aggressive behavior, severe anxiety, social avoidance, short stature, scoliosis, high pain tolerance, dysmorphic features, mild micrognathia, joint hypermobility, small hands, brachydactyly with hypoplastic distal phalanges of all fingers and very short fourth and especially fifth metacarpals and metatarsals.	HDAC8 c.943T>A (p.Trp315Arg) {het-DN} [NM_018486.2]	Cornelia de Lange syndrome (300882); XLD	LP
P20	14-yo/M	Quad (with affected 4 yo brother and affected father)	Proband: Primary microcephaly, seizures, ID, attention deficit Affected Brother: Primary microcephaly and seizures, ptosis, astigmatism, nystagmus, epicanthal folds, posterior parietal hair whorls, a large incisor, smooth philtrum, and a single transverse palmar crease. Father: Microcephaly	KIF11 c.308+1G>A, {het-pat} [NM_004523.3]	Microcephaly with or without chorioretinopathy, lymphedema, or intellectual disability (152950); AD	VUS

Aneuploidies and UPD						
ID	Age/gender	Family structure^a^	Phenotype	Genomic variant(s)	Associated condition (phenotype MIM number)	Classification^b^
P21	4.5-yo/F	Duo	Global DD, ataxia, dysmorphic facies, facial asymmetry while crying, feeding difficulties, aggressive behavior	Depth of 4× on p-arm of chr18	Tetrasomy 18p (614290)	P
P22	1.5-yo/F	Trio	Multiple congenital anomalies, hypomelanosis of Ito	Depth of 2.47× on chr14^c^	Mosaic trisomy 14	P
P23	4-yo/F	Trio	Global DD, dysmorphic facies, broad- based ataxic gait	UPD of chr15 {pat}	Angelman syndrome (105830)	P

CNVs and derivative chromosomes						
ID	Age/gender	Family structure^a^	Phenotype	Genomic variant(s) {inheritance}	Associated condition (phenotype MIM number)	Classification^b^
P24	11-yo/M	Duo	DD, ID, FTT, dysmorphic facies, cleft palate, tapered fingers, PDA, hypoplastic nails	6.6 Mb loss at 2q36.1-q37.3, mosaic (chr2:236478472-243048854)^d^ 2.7 Mb gain at 3q29, mosaic (chr3:195106447-197846145)^d^	Derivative chromosome 2, including 2q37 microdeletion syndrome	P
P25	5-yo/F	Duo	DD, ID with expressive language delay, behavioral issues, joint laxity, loose skin, easy bruising, low tone, dysmorphic facies, bifid uvula, two supernumerary nipples, diastasis recti, overlapping toes, flat feet, a single transverse palmar crease, prominent finger pads on her right hand, 4th finger clinodactyly, decreased fetal movement in prenatal stage with dorsiflexion of the feet and fisted hands at birth	510.8 kb loss at 21q22.3 (chr21:47590586-48101334) 34.4 Mb gain at 3p26.3-p22.3 (chr3:60000-34461438)	Derivative chromosome 21, including partial trisomy 3p syndrome	P
P26	9.5-yo/M	Duo	Growth restriction with microcephaly, ID, absent speech, hyperactivity, dysmorphic facies, seizures	15.5 Mb loss at 6q21-22.31 (chr6:109324789-124836619) {unk} 2.5 Mb loss at 6q22.33-23.2 (chr6:129969121-132499298) {unk} 1.95 Mb gain at 11p15.4 (chr11:8548056-10497905) {unk}	Acro-cardio-facial syndrome (6q21-6q22.1 deletion); complex chromosomal change Complex chromosomal change Complex chromosomal change	P VUS VUS
P27	15-yo/M	Duo	DD, ID, recurrent infections, cryptorchidism, mild hearing loss, mild autistic behavior, crowded teeth, micrognathia, second toe overlaps the first toe on both feet	3.48 Mb gain at 1q22-q23.2 (chr1:155709113-159191078) {unk}	Disease association NOS	VUS-LP
P28	3-yo/F	Trio	DD, FTT, PDA with pulmonary hypertension, poor growth with microcephaly, dysmorphic facies, including bilateral 5th finger clinodactyly, hypoplastic nails, protuberant and anteverted ears, short and broad hands and feet, excessive sweating, feeding difficulties	3.78 Mb loss at 15q26.3 (chr15:98615561-102400033) 15.21 Mb gain at 13q32.2q34 (chr13:99892724-115108414)	Derivative chromosome 15	P
P29	9-yo/M	Trio	ID, hypotonia, growth deficiency, microcephaly with sagittal and metopic craniosynostosis, two anterior hair whorls, large malformed ears with narrow canals, dysmorphic facies, bilateral 2,3,4 campodactyly, bulbous toes, a short left fourth metatarsal, contracted knees, one kidney smaller than the other, cryptorchidism	18.3 Mb loss at 1p36.12-36.32 (chr1:4436802-22782007) {DN}	1p36 deletion syndrome (607872)	P
P30	4.5-yo/F	Trio	ID, DD, aggressive behavior, anxiety, macrocephaly, dysmorphic facies, hypertrichosis, large hands, large ears, MRI findings of an increase in extra-axial space and dilatation of the Virchow–Robin space	2 Mb loss at 5q35.2-35.3 (chr5:175470000-177450000) {DN}	Sotos syndrome (117550)	P
P31	20-yo/M	Trio	Expressive speech delay, learning disabilities, hypertrophic cardiomyopathy, telangiectasia, facial flushing, acroparesthesia, joint pain, headaches	5.1 Mb gain at 8p23.1 (chr 8: 7153587-12245784)^e^ {DN}	8p23.1 duplication syndrome	P
P32	1-yo/F	Trio	​Gross motor DD, prenatal growth restriction, FTT, striking generalized hypotonia with head lag, ASD with pulmonary insufficiency, mild nasopharyngeal reflux, dysmorphic facies, bilateral shoulder dimples, small hands with prominent fetal pads, bilateral single transverse palmar creases, tapered fingers, bilateral fifth finger clinodactyly, small and puffy feet	5.76 Mb loss at 14q32.31-14qter (chr14:101522804-107289470) {DN}	14q32.3 terminal deletion syndrome	P
P33	10.5/F	Trio	DD, ID, hypotonia, dysmorphic facies, partial 2–3 and 3–4 syndactyly of the hands, astigmatism, anterior crossbite, sleep apnea	2.4 Mb loss at 1q42.2-42.3 (chr1:234117880-236536349) {DN}	1q4 deletion syndrome	P
P34	5.5-yo/M	Trio	DD, bilateral club feet, inguinal and umbilical hernias, aplasia cutis congenita, spina bifida occulta, dysmorphic facies, long fingers, and small patellas which are dislocated to the left	9.86 Mb loss at 5q23.2-q31.1 (chr5:124864529-134720575) {DN}	5q22-q31 deletion syndrome	P
P35	1-yo/M	Trio (with affected father)	FTT, dysmorphic facies, mild retrognathia, low set ears, small hands with brachydactyly, fifth finger clinodactyly, and persistent fetal pads, infantile acne, large hypopigmented spot on abdomen	2.3 Mb loss at 1q24.2-25.1 (chr1:170777501-173113232) {pat}	1q24-25 deletion syndrome	VUS-LP
P36	10-yo/M	Trio	DD, behavioral issues, feeding difficulties, hyperextensible and buckling phalanges, dysmorphic facies	26.3 kb loss at 14q32.3, mosaic (chr6:101261679-101288013)^f^ {DN}	Kagami–Ogata syndrome (608149)	VUS
P37	11-yo/F	Quad	ID, microcephaly, absent speech, ataxia, aggressive and self-injurious behavior, dysmorphic facies	3.02 Mb loss at 2p25.3 (chr2:11314-3033976) 7.30 Mb gain at 16q23.3-24.3 (chr16:82865402-90163542)	Derivative chromosome 2	P
P38	1.5-yo/M	Quad with affected maternal half-brother	ID, ASD, prominent fetal pads, long fingers, multiple ear tags, bilateral ear pits, low hairline, microcephaly, non-verbal, non-ambulatory	3.5 Mb gain at 22q11.1-q11.21 (chr22:16849364-20311389) 18.3 Mb gain at 11q23.3qter (chr11:116684536-134945187)	Emanuel syndrome (609029)	P

Multiple variant types implicated in primary molecular diagnoses						
ID	Age/gender	Family structure^a^	Phenotype	Genomic variant(s) {molecular state-inheritance}	Associated condition (phenotype MIM number)	Classification^b^
P39	14-yo/F	Trio	DD including cognitive deficiency and lack of speech, growth deficiency, epilepsy, behavioral issues including autism and self-injurious tendencies, lack of secondary sexual characteristics, coarse facies, long tapered fingers, thick lips, over-folded ears, broad-based and duck-footed gait, lactose intolerance, constipation	SCN2A c.224C>G (p.Ser75Ter) {het-DN} [NM_001040142.1] KISS1R c.233A>G (p.Asn78Ser) {hem-mat} [NM_032551.4] 358.7 kb loss at 19p13.3 (chr19:916637-1275315) – Includes full-gene deletion of KISS1R and STK11 {DN} Incidental finding: CHEK2 c.349A>G (p.Arg117Gly) {het-mat} [NM_007194.3]	SCN2A-related disorders (607745); AD Isolated GnRH Deficiency (614837); AR Isolated GnRH Deficiency (614837); AR and Peutz–Jeghers syndrome (175200); AD as secondary finding CHEK2-related cancer susceptibility (609265); AD	P VUS P LP
P40	1-yo/M	Trio	Joint contractures, short neck, dysmorphic facies, bilateral cryptorchidism, micrognathia, hypoplastic corpus callosum	ECEL1 c.793C>T (p.Gln265Ter) {het-pat} [NM_004826.2] ECEL1 c.110_155delTCCCGTTGGGC GCTGCGCGCAGCGCCACCGGGGCCCGGTCCGGGCT (p.Phe37CysfsTer151) {het-mat}^g^ [NM_004826.2]	Distal arthrogryposis, type 5D (615065); AR	LP LP
P41	9-yo/M	Trio with mother affected with myopathy	Scapular winging, severe muscle weakness, long and myopathic facies, clinical diagnosis of Down syndrome Mother: Muscle weakness and abnormal muscle biopsy	ACTA1 c.821C>T (p.Ala274Val) {het-mat} [NM_001100.3] Depth of 3× on chr 21	Nemaline myopathy (161800); AD Trisomy 21/Down syndrome (190685)^h^	LP P

*M* male, *F* female, *SNV* single nucleotide variant, *indel* insertions and deletions, *UPD* uniparental disomy, *CNV* copy number variant, *P* pathogenic, *LP* likely pathogenic, *VUS* variant of unknown significance, *DD* developmental delay, *ID* intellectual disability, *FTT* failure to thrive, *PDA* patent ductus arteriosus, *ASD* atrial septal defect, *ADHD* attention deficit hyperactivity disorder, *AR* autosomal recessive, *AD* autosomal dominant, *XL* x-linked, *XLD* x-linked dominant, *het* heterozygous, *hom* homozygous, *hem* hemizygous, *mat* maternal, *pat* paternal, *unk* unknown, *DN* de novo, *NOS* not otherwise specified

^a^All family members sequenced for family-based analysis were unaffected unless otherwise specified in the table.

^b^Classification per ICSL laboratory interpretation, following the general framework of the ACMG Guidelines^19,20^

^c^ICSL cWGS purity estimate for trisomy 14 is 47%, orthogonally confirmed at 51% purity by external lab via chromosomal microarray;

^d^ICSL cWGS purity estimate for derivative chromosome 2 is 64%, orthogonally confirmed at 63% purity by external lab via chromosomal microarray

^e^Associated syndrome explains only part of proband’s phenotype

^f^Variant falls outside ICSL test definition and was not confirmed by an external clinical laboratory

^g^Orthogonally confirmed by external lab via targeted sequencing

^h^Previously clinically diagnosed and cytogenetically confirmed with 47, XY + 21 karyotype

**Fig. 2:**
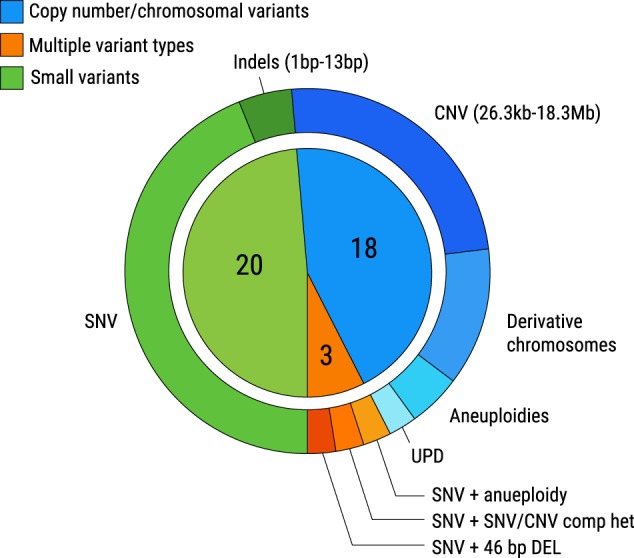
Proportion of variant types observed in cases where molecular diagnoses were achieved. Number of probands with small variants (including SNVs and indels), copy number/chromosomal variants (including CNVs, derivative chromosomes, aneuploidies, and UPD), and multiple variant types (SNVs and another variant type in a single case) are noted. SNV: single nucleotide variant, indel: insertions and deletions, UPD: uniparental disomy, CNV: copy number variant

A range of variant types were observed in the 41 cases in which primary molecular diagnoses were achieved. These included SNVs (*n* = 18; 43.9%), CNVs ranging from 26 kb–18 Mb (*n* = 10; 24.4%), multiple terminal CNVs suggestive of derivative chromosomes (*n* = 5; 12.2%), aneuploidies (*n* = 2; 4.9%), absence of heterozygosity (AOH) consistent with uniparental isodisomy (UPD) (*n* = 1; 2.4%), indel (*n* = 2; 4.9%), a compound heterozygous variant pair involving multiple variant types (*n* = 1; 2.4%), a dual diagnosis of SNV and aneuploidy (*n* = 1; 2.4%), and one case with at least four molecular diagnoses including a SNV and a compound heterozygous pair involving multiple variant types (*n* = 1; 2.4%), as summarized in Fig. 2.

No molecular diagnosis was achieved in 19 cases. In four negative cases, birth injury or teratogenic exposures including monozygotic twin-related injury, valproic acid embryopathy, fetal alcohol syndrome, and Zika virus-related embryopathy, were noted by the clinician team as among the differential diagnoses. In one negative case, the proband is suspected to have a genetic disorder involving mosaicism given her hyperpigmented and hypopigmented patchy skin lesions on the legs and skeletal asymmetry including hypoplasia of the left humerus and shoulder girdle. In some negative cases (*n* = 4; 6.7%), variants of interest were reported for clinical consideration (Table 3). These probands are not considered to have received molecular diagnoses, but clinicians have characterized these variants of clinical interest as either possibly contributory (*n* = 3) or secondary (*n* = 1) molecular findings in relation to the proband’s phenotype.

**Table 3:** Reported variants of clinical interest reported by variant type

ID	Age/gender	Family structure^a^	Phenotype	Genomic variant(s) {molecular state- inheritance}	Associated condition (phenotype MIM number)	Classification^b^
P42	6-yo/F	Duo analysis	Congenital inflammatory myopathy, hypotonia, absent reflexes, muscle pain, motor delay	1.6 Mb gain at 16p13.11 (chr16:14800000-1640000)	16p13.11 microduplication syndrome	P
P43	3-yo/M	Trio analysis	DD, bilateral cryptorchidism, macrocephaly, mild hepatosplenomegaly, frequent respiratory infections, dysmorphic facies	USP7 c.1033G>A (p.Glu345Lys) {het-DN} [NM_003470.2]	Disease association NOS	VUS
P44	5-yo/F	Trio	DD, speech delay, post-natal growth deficiency, microcephaly, dysmorphic facies, low frontal hair line with a widow’s peak, synophrys, hirsutism, prominent fetal pads, bifid distal phalanx of the left second toe with small nails, hypoplasia of the labia majora and labia minora with possible absence of clitoris with an anterior anus and ano-vaginal fistula.	VPS13B c.3767T>G (p.Met1256Arg) {het-mat} [NM_017890.4] VPS13B c.3811A>T (p.Thr1271Ser) {het-pat} [NM_017890.4] VPS13B c.7787C>T (p.Ser2596Phe) {het-pat} [NM_017890.4]	Cohen syndrome (216550); AR	VUS VUS VUS
P45	5-yo/M	Trio	Global DD, feeding difficulties, behavioral issues including hyperactivity and aggression	EP300 c.1528+3_1528+10delAAGTTTGT {het-DN} [NM_001429.3]	Rubinstein-Taybi syndrome (613684); AD	VUS

*M* male, *F* female, *P* pathogenic, *VUS* variant of unknown significance, *DD* developmental delay, *AR* autosomal recessive, *AD* autosomal dominant, *het* heterozygous, *mat* maternal, *pat* paternal, *DN* de novo, *NOS* not otherwise specified

^a^All family members sequenced for family-based analysis were unaffected unless otherwise specified in the table

^b^Classification per ICSL laboratory interpretation, following the general framework of the ACMG Guidelines^20^

Surveys assessing the clinical utility of cWGS results were completed by the clinical team for all 60 probands. Of the 41 probands who received a molecular diagnosis, cWGS results produced new clinical diagnoses in 80.5% of cases (*n* = 33) and confirmed clinical diagnoses in 19.5% of cases (*n* = 8). A change in the proband’s clinical course due to cWGS findings was reported in 48.8% of cases (*n* = 20). These included referrals to specialists to assesses for co-morbidities (*n* = 8; e.g., neurology, ophthalmology, audiology) or for imaging or functional testing (*n* = 6; e.g., renal ultrasound, brainstem auditory evoked response test, echocardiogram, electrocardiogram, electroencephalogram). Muscle biopsy was avoided in three probands who received molecular diagnoses, reducing potential morbidity and clinical resource burden. Some findings (*n* = 3) led to additional clinical investigations related to the molecular diagnosis, including abnormal eye movements further investigated for possible seizures and examination of muscle tone in association with progressive spastic paraplegia. One family received information regarding the creation of an augmentative communication system for a child with Angelman syndrome, a condition in which there is typically poor prognosis for the development of expressive verbal language. One patient was transitioned to palliative care after molecular diagnosis of neuronal ceroid lipofuscinosis. Finally, current or future screening for malignancies and/or tumors/polyps was discussed for the proband with a deletion encompassing exons 1–10 of the *PMS2* gene (P6), for the proband with an entire gene deletion of *STK11* (P39), and for the proband and mother with a pathogenic *BRCA2* variant (P12) identified by cWGS as secondary findings.

Among cases where no molecular diagnoses were achieved, cWGS was reported to be helpful to the proband’s clinical care in at least six probands and their families. In two cases where the proband’s brain imaging suggested possible leukodystrophy, cWGS was reported to be helpful to clinicians in communicating a significantly reduced likelihood for suspected specific diagnoses. In three negative cases, clinicians were motivated to expedite additional clinical work-up to further investigate the proband’s phenotype, including ophthalmology exams and a muscle biopsy. For one proband (P43) with no molecular diagnosis, a variant of clinical interest was submitted to GeneMatcher^12^ and yielded contact from three institutions and enrollment in a clinical study of patients with suspected causative variants in the *USP7* gene.

Post-test genetic counseling was modified for 49 families (100% of probands with molecular diagnoses; 81.7% of the cohort) following cWGS results. Among cases with molecular diagnoses, the most frequently cited reason for the change in post-test counseling was the ability to share information about recurrence risk/options for preconception testing or prenatal diagnosis (*n* = 37) and prognostic indications (*n* = 21). Among negative cases, the most frequently cited reasons for change in post-test counseling was ability to counsel regarding decreased likelihood of genetic conditions (*n* = 3).

## Discussion

In this cohort summary, we have shown that deployment of cWGS as a first-tier test in a resource-limited clinic is able to identify diverse, clinically significant genomic variation in the majority (68%) of probands with suspected genetic disease, almost half of whom had a change in clinical course due to the molecular findings. The ability to detect a wide breadth of disease-causing genomic variation via a single test offers potential benefit for a variety of patients, including those with a broad differential diagnosis, disease-causing variants of different types, and/or with dual diagnoses. For example, in an 11-year old male proband (P40), a paternally inherited SNV and a maternally inherited 46-bp deletion in the *ECEL1* gene were identified simultaneously using cWGS, consistent with autosomal recessive distal arthrogryposis. A dual diagnosis was obtained in a child with clinically diagnosed atypical Down syndrome (P41), which included long myopathic facies, scapular winging, tapered calves, infantile spasms, severe muscle weakness, and hypotonia, who was found to harbor both trisomy 21 and a maternally inherited, likely pathogenic *ACTA1* SNV associated with nemaline myopathy. Four molecular diagnoses were reported in a 14-year-old female with a phenotype including developmental and cognitive delays, epilepsy, growth deficiency with lack of secondary sexual characteristics, and dysmorphic features (P39), with contribution from both SNVs and a CNV. Two of these diagnoses were considered primary diagnoses, consistent with the patient’s current reported phenotype, while the other two diagnoses were secondary or incidental findings. This individual may not have received all molecular diagnoses by conventional clinical serial testing strategies, even in a resource-rich area, as the likelihood of continuing the testing process is low once an initial finding is obtained.

The detection of CNVs (*n* = 15), aneuploidies (*n* = 2), and dual diagnoses involving copy number variation (*n* = 3) accounted for 48.8% (20/41) of diagnostic findings. This likely reflects population-specific resource limitations, including reduced access to cytogenetic testing and very limited access to chromosomal microarray testing prior to cWGS. It is possible that many of these variants would have been detected by microarray, but the cost of microarray remains prohibitively expensive for many families served in this resource-limited dysmorphology clinic. We therefore opted to utilize donated cWGS as a first-line test, as opposed to a reflex for patients with non-diagnostic microarray testing, so as not to exclude families with financial barriers to accessing microarray. As the cost of WGS continues to decline, future studies may directly assess the cost-effectiveness and clinical implications of first-tier WGS testing against reflexive WGS or exome sequencing after non-diagnostic microarray.

There is also accumulating evidence that WGS out-performs microarray in both sensitivity and specificity. For example, a prospective study of patients referred for pediatric genetics evaluation showed a four-fold increase in diagnostic yield with WGS over chromosomal microarray alone.^10^ These data also highlight the distinctive diagnostic benefits of WGS, including the detection of single or multiple molecular diagnoses resulting from both pathogenic SNVs and CNVs in a single test. Additionally, a recent systematic evaluation of CNV calling in the context of a cWGS testing found that cWGS showed greater sensitivity for pathogenic CNVs across all size ranges.^13^ This cohort also provides some examples of CNVs detected by cWGS that may have been missed by other approaches, including a mosaic 26.3 kb deletion at 14q32.2 that was detected in a 9-year-old proband (P36) with developmental delay, neonatal respiratory and feeding difficulties, and distinctive features (full cheeks, myopathic facies, anteverted nares, and hyperextensible fingers).^13^ This locus contains two differentially methylated regions (DMRs) involved in the imprinted regulation of MEG3. The maternal deletion of these regions has previously been associated with Kagami–Ogata syndrome^12^ (MIM 608149) and shows considerable phenotypic overlap with P36. Given the size and location of this finding, it may not have been detected by some clinically available microarray or exome tests.

Clinicians’ survey responses endorsed the clinical utility of cWGS, with changes reported in both the proband’s clinical management and post-test genetic counseling. Although a change in post-test genetic counseling was expected for most probands with molecular diagnoses, altered counseling was reported for over three quarters of the cohort, including those that did not receive a molecular diagnosis. In these cases, the negative cWGS results did not support the suspicion of genetic diagnoses and modified the provision of counseling regarding decreased likelihood of genetic conditions. In families with increased screening recommendations due to secondary findings, additional financial burden placed on the family was considered by the clinical team. Modified screening may be recommended (e.g., colon cancer screening to include CBC for anemia and fecal occult blood test initially, with follow-up colonoscopy if indicated after these initial tests) to help mitigate this burden.

Overall, these results suggest that cWGS as a first-tier test can achieve a wide range of molecular diagnoses which influence care, even in a resource-limited setting. If cWGS can be deployed at reasonable cost, this may be a helpful testing strategy in resource-limited populations where a serial testing approach would be particularly onerous. Further investigation is required to understand if diagnostic yield can be further increased by the detection of additional variant types (e.g., mitochondrial deletions, smaller copy number variation, repeat expansions). Additional study is also required to understand if these findings will be applicable to other pediatric populations in resource-limited settings, particularly given the detailed case selection performed by the dysmorphology specialist clinical team. Since the completion of this cohort summary, the iHope Program has expanded to partnerships with a total of 10 external clinical sites including continued support of cWGS test donation to the Foundation for the Children of the Californias/Hospital Infantil de Las Californias. Ongoing analysis of cWGS data continues for consented probands with no molecular diagnoses through an Illumina IRB-approved research study.

## Methods

### Patient selection information

Clinical whole genome sequencing tests were philanthropically provided through Illumina’s iHope Program to patients seen at the dysmorphology clinic held monthly at the Hospital Infantil de Las Californias. Case acceptance criteria for cWGS testing through the iHope Program included: (1) referral from pediatrician to the dysmorphology clinic for evaluation of congenital anomalies and/or suspected genetic disorder; (2) clinical genetics evaluation to include medical history, three generation pedigree, and physical examination with attention to major and minor malformations; and (3) inability of the family to receive appropriate genetic testing due to financial hardship. Patients were ascertained concurrently and through chart review of previously evaluated individuals (Fig. 1). Families eligible for the iHope Program were invited to participate in a “Genome Day”: a visit to the dysmorphology clinic in Mexico that included an updated clinical examination, facilitation of informed consent, and blood draws of all consented family members. Informed consent for cWGS testing was facilitated in Spanish and obtained in accordance with the rules and standard operating procedures of the Illumina Clinical Services Laboratory and the Hospital Infantil de las Californias, including a review of the possibility to receive secondary findings related to highly penetrant genetic disease per recommendations from the American College of Medical Genetics and Genomics (ACMG).^13^ Whole-blood samples were submitted for the TruGenome™ Undiagnosed Disease cWGS test at the Illumina Clinical Services Laboratory, a CLIA-approved and CAP-accredited laboratory located at Illumina Inc in San Diego, California. Subsequent retrospective analysis of patient data from this cohort was conducted in accordance with requirements of approval by the Western Institutional Review Board.

### TruGenome™ undiagnosed disease cWGS test

The TruGenome™ Undiagnosed Disease test is designed to detect and report on SNVs, small indels, CNVs, and mitochondrial DNA SNVs that impact genes which have an established association to genetic disease. Whole genome sequencing was performed on DNA extracted from whole-blood and prepared for next-generation sequencing using the Illumina TruSeq™ PCR-free kit. Samples were sequenced on the Illumina HiSeq™ X™ system with paired-end 150 base pair reads at the Illumina Clinical Services Laboratory in San Diego, California, USA. The data were aligned^14^ according to build 37 of the Human Reference Genome (http://www.ncbi.nlm.nih.gov/projects/genome/assembly/grc/human), and analyzed using the Strelka^15^ caller for SNVs and Canvas^16^ caller for CNVs.

Genomes were sequenced to an average of ≥30 fold coverage. The range of mean depth of coverage across this cohort of 167 samples was 32× to 42×. Interpretation was performed for SNVs and indels that fall within 15 bp of a RefSeq exon boundary. Based on the quality filters and through the analysis of an extended, multi-generation family set (Platinum Genomes),^17^ sensitivity for SNVs is 98.9% and sensitivity for insertions up to 31 bases and deletions up to 27 bases is 80–85%. This assay has the capability to detect copy number events greater than 10 kb, however sensitivity was only assessed for events greater than 20 kb and was found to be approximately 85%.^13^ CNV interpretation was limited to events that either overlapped an exon or had a boundary that was within 1 kb upstream or downstream of an exon. Mitochondrial SNVs detected at an allele fraction greater than or equal to 3% were interpreted for pathogenicity for a samples analyzed during and after June 2017; however, percentage of heteroplasmy was not a component of the validated cWGS laboratory test at the time of this testing. The sensitivity metrics reported above reflect the most current test definition, and can range within 1–2% for some samples due to test development and validation of evolving clinical laboratory assays over time. Variants of interest identified by cWGS and reported to the clinicians which were not within the clinical test definition were orthogonally tested through external clinical laboratories, if possible.

### Variant assessment

Variants of interest were identified based on consideration of population allele frequency, variant consequence, evolutionary conservation, occurrence in a gene with an established gene–disease relationship, occurrence in a gene whose disease association overlaps with the patient’s reported phenotype, and inheritance mode, as appropriate. A directed query of the Online Mendelian Inheritance In Man (OMIM) database was performed for each case using terms reflecting the proband’s phenotype. The resultant gene list was used to prioritize variants from the family-based analysis. Variant interpretation for SNVs and CNVs was performed according to the ACMG guidelines for variant classification^18,19^ and a clinical report was issued for the proband to include genomic findings of potential clinical significance. Ancillary reports for secondary findings and a pharmacogenomics screen were also provided for all individuals who received cWGS.

### Assessment of clinical care in the context of cWGS findings

To investigate the clinical impact of cWGS findings, semi-structured surveys designed to assess the impact of cWGS results on clinical care and post-test genetic counseling modifications were administered to the clinical team (Supplementary Table 1). The survey is comprised of multiple-choice questions as well as free-text explanations. Survey responses were utilized to report on the following: (1) if the cWGS test contributed to the proband’s diagnosis; (2) if a change in clinical course was recommended or may be considered based on the cWGS results and (3) if any additional clinical actions resulting from the cWGS test occurred. A contribution to the proband’s diagnosis was determined if clinicians reported that the cWGS result(s) produced a new diagnosis or confirmed a clinical diagnosis. A change in clinical course is defined as a change in management and/or reports of additional clinical testing or follow-up for the proband, which was directly related to the cWGS result. Results from multiple-choice questions were tabulated, and free-text explanations were used to provide additional details and examples.

The clinician team stratified probands into two groups based on phenotype characteristics: suspected pattern of malformation and primary neurologic presentation. Phenotypes classified as suspected patterns of malformation include those with multiple organ systems affected, dysmorphic features, and other variable clinical presentations expected to indicate a pleiotropic syndrome. The classification of neurological presentation included generally non-dysmorphic probands in whom the primary phenotype was neurological in origin. This enabled the team to determine if there were differences in molecular diagnostic success between these two broad phenotypic groups in this cohort. Statistical comparison of the diagnostic yields between different phenotype groups was performed using a Fisher’s exact test.

## Supplementary information

iHope Clinical Utility Survey

## Data Availability

The data that support the findings of this study are available from Illumina Clinical Services Laboratory, but restrictions apply to the availability of these data due to them containing sensitive information that could compromise patient privacy/consent and so are not publicly available.

## Supplementary information

**Supplementary information** accompanies the paper on the *npj Genomic Medicine* website (10.1038/s41525-018-0076-1).
