# Supplementary material for: Clinical whole genome sequencing as a first-tier test at a resource-limited dysmorphology clinic in Mexico
Source: NPJ Genom Med. 2019 Feb 14;4:5. doi: 10.1038/s41525-018-0076-1 (PMC6375919; doi:10.1038/s41525-018-0076-1)
Supplement: Supplementary file 1 — iHope Clinical Utility Survey [file 41525_2018_76_MOESM1_ESM.pdf]

iHope

Barcode Number:

Clinical Utility Form

1. Diagnoses prior to submission of sample for whole genome sequencing (WGS):

2. Diagnoses after receipt of WGS

3. WGS (please select the best response):

   ☐ Confirmed the clinical diagnosis

   ☐ Produced a new diagnosis

   ☐ Gave a possible diagnosis

   ☐ Ruled out a suspected diagnosis

   ☐ Did not contribute to the diagnosis

   ☐ Other, please specify:

   ☐ NA

4. If WGS confirmed or produced a diagnosis, was confirmatory testing then performed?

   ☐ YES

   ☐ NO

   ☐ NA

5. Did WGS change the management of the patient?

☐ YES

☐ NO

☐ NA

If no, skip to question 6. If yes, mark all that apply:

☐ Condition specific management (specific therapy targeted to the diagnosis)

Specify therapy:
Specify therapy:
Specify therapy:

☐ Condition Specific Supportive Interventions (supportive care informed by a better understanding of the diagnosis)

Specify supportive care:
Specify supportive care:
Specify supportive care:

☐ Palliative or End of Life Care (palliative care informed by an improved understanding of the prognosis)

Specify type of palliative care:
Specify type of palliative care:
Specify type of palliative care:

6. Did the WGS result change the post genetic consultation?

☐ YES If YES, describe how?

☐ NO

☐ NA

7. The results of the WGS led to additional clinical testing (check all that apply)

☐ Specialty consultation Specify:
☐ Physiological testing Specify:
☐ Imaging Specify:
☐ Laboratory Specify:
☐ None
☐ NA

8. Please provide any additional detail:
